# Muscular Strength of Upper and Lower Limbs and Self-Esteem in Chilean SchoolChildren: Independent Associations with Body Composition Indicators

**DOI:** 10.3390/ijerph18020361

**Published:** 2021-01-06

**Authors:** Cristian Cofre Bolados, Gerson Ferrari, Mónica Suárez-Reyes, Daiana Quintiliano Scarpelli Dourado, Helen Diaz-Peña, Tito Pizarro

**Affiliations:** 1 Laboratorio de Ciencias de la Actividad Física, el Deporte, y la Salud, Facultad de Ciencias Médicas, Universidad de Santiago de Chile, USACH, Santiago 7500618, Chile; gerson.demoraes@usach.cl (G.F.); monica.suarez@usach.cl (M.S.-R.); tito.pizarro@usach.cl (T.P.); 2 Carrera de Nutrición y Dietética, Facultad de Medicina-Clínica Alemana, Universidad del Desarrollo, Santiago 7610658, Chile; dquintiliano@udd.cl; 3 Pediatric Oncology Department, Clínica Dávila, Santiago 8431657, Chile; helendiaz@davila.cl

**Keywords:** muscular strength, self-esteem, body mass index, body fat

## Abstract

The aim of this study was to analyze the relationship between muscular strength from upper and lower limbs with self-esteem among Chilean schoolchildren, drawing independent associations with body composition indicators. The sample consisted of 1078 schoolchildren. The muscular strength of the upper and lower limbs was evaluated using a digital dynamometer and long jump performance. The general strength index was calculated based on Z-score values. Rosenberg’s test was used to determine the level of self-esteem of participants. Body fat and body mass index were employed as body composition indicators. Boys had significantly more upper and lower strength, and a general strength index higher than girls (11.7 vs. 10.6; 109.7 vs. 97.4; 0.19 vs. −0.24, respectively). For boys and girls combined, there were no significant associations between all muscular strength variables and self-esteem. In boys, upper and lower limb strength was positively associated with self-esteem. In girls, no association between muscular strength and self-esteem was found. In both sexes, the general strength index was not associated with self-esteem. Strategies and programs that promote mental health and muscular strength among schoolchildren, specifically in boys, are needed.

## 1. Introduction

Mental health problems cause a huge public health burden that affects 10–20% of children and adolescents worldwide, and account for a large portion of the global burden of disease [1]. Childhood is a fundamental period of life when numerous psychological changes occur, and thus psychological health at this stage may be a factor for later stages in life [2]. Psychological health is understood as the absence of psychological distress with the occurrence of psychological well-being, which is defined as positive affective states and conducting oneself with ideal effectiveness in personal and social life (e.g., self-esteem) [3]. Self-esteem is a critical aspect that affects the psychosocial stability of children, and is normally linked to a specific health status [4].

Lower levels of self-esteem have been associated with psychopathological symptoms such as anxiety [5], depression, loss of hope, suicidal tendencies [6], and procrastination; in addition, aggressive behavior [7], antisocial conducts [8], and bullying [9] are often observed. A factor to be noted is that children with low self-esteem present poor physical health, and individuals that teach children with decreased self-esteem are at more risk of exhibiting unhealthy habits and low self-esteem [10].

A systematic review showed that physical fitness has a beneficial effect on health outcomes, such as a lower risk of cardiovascular disease, diabetes, hypertension, cancer, and obesity [11,12,13]. Additionally, it may produce psychological benefits as well [14]. Growing literature suggests that physical fitness can improve mental health, including self-esteem [15,16]. A school-based physical fitness intervention for young people in Chile led to better self-esteem [17]. Similarly, muscular strength was related to better self-esteem [18], as well as a reduced risk of any future psychiatric diagnosis and suicide mortality [19]. Likewise, the level of muscular conditioning in childhood and adolescence seems to last until adulthood [18].

Obese children may have less experienced self-esteem than their peers with a normal weight [20,21]. Childhood obesity has rapidly increased over the last decades in Chile, with it now being one of the highest in the world [22,23], with more than 50% overweight and obese children [22]. However, to the best of our knowledge, some studies have demonstrated an association between muscular strength and self-esteem of schoolchildren when obesity indicators are controlled for. Thus, the aim of this study is to analyze the association of muscular strength of upper and lower limbs with self-esteem among Chilean schoolchildren with an average age of 9 years, drawing independent associations with body composition indicators.

## 2. Materials and Methods

### 2.1. Study Design and Participants

This cross-sectional study is part of the Promotion of Scientific and Technological Development (Fondo Nacional de Desarrollo Científico y Tecnológico—FONDEF) project “Development, scaling, and validation of an integrated system for school interventions on diet, physical activity, and community environment”. The scope of the study consisted of all students enrolled in primary schools from the southern area of Santiago (*n* = 6.308). The sample corresponded to second to fourth grade students (mean age of 9 years) from seven schools in the municipalities from the “Ciudad Sur” municipalities association of the Metropolitan Region of Chile (El Bosque, Pedro Aguirre Cerda, San Joaquín, San Ramón, La Granja, and Lo Espejo). The data were obtained from the census conducted in March 2019, as well as information provided by the Municipal Education Departments of each municipality. Stratification, proportionality, and randomization techniques were employed to establish a representative sample (sample error 0.04; IC = 95.5%). Stratified random sampling with proportional affixation was used to ensure homogeneous representation of the sample. The strata considered were grade (second, third, and fourth grades from primary school) and sex (male and female). To achieve representativeness, two schools were selected per municipality, with a final sample of 1078 children (598 boys and 480 girls).

The research was conducted in accordance with the 1975 Declaration of Helsinki. In addition, the work was approved by the Ethics Committee of Universidad de Santiago de Chile (record number 187/2019). Likewise, the legal guardians of students participating in the survey, as well as the school community, were informed of the nature of the study prior to obtaining their informed consent. The research team was present during the application of the instruments in order to ensure their correct implementation. Students completed the questionnaire anonymously, ensuring confidentiality, and performed the physical condition tests during their regular physical education class and sports workshops. Tests were conducted by physical education and sports sciences professionals who were previously trained in the application of the tests, and were supervised by the expert researchers during all of the measurements.

### 2.2. Muscular Strength

Maximum hand grip strength was measured using a digital dynamometer Jamar^®^ PC-5030 (Jamar Dynamometer, Lafayette, IN, USA), which has a measurement range from 5 to 100 kg and a precision of 100 g, through two alternated attempts per hand, a standardized position for the foot, and arms parallel to the body without touching it. A long jump with feet together was used as a measure to determine the maximum distance traveled by the lower limbs in two attempts. With the results of these tests, the general strength index (Z-score) was calculated by combining the standardized values of hand grip strength (kg) and long jump (cm). Each of these variables was standardized as follows: Z-score = ((value-mean)/ standard deviation). The general strength index was calculated as the mean of the two standardized scores (hand grip strength and long jump). The Z-scores for the muscular strength index in children were the same as those used in the literature [24,25]. The average of the two tests converted (z-score) was used to establish a single variable, as proposed by Garcia-Artero [26].

### 2.3. Self-Esteem Assessment

Rosenberg’s test was used to determine the level of self-esteem of participants [27]. The reliability and validity of the questionnaire were evaluated; as a result, all of the items included exhibited test–retest reliability intraclass correlations >0.50. This questionnaire was comprised of 10 items rated through a four-point Likert scale, where 1 = “Strongly disagree” and 4 = “Strongly agree”. Items 1, 3, 4, 7, and 10 were written in an affirmative way, while items 2, 5, 6, 8, and 9 were worded using negation. The scores ranged from 10 to 40 points, with higher values indicating higher self-esteem. Self-esteem was classified as high (≥30 points), medium (26–29 points), or low (≤25 points) [28].

Finally, scores were added to determine the level of self-esteem. This instrument was adapted to Spanish [29]. The translation of the instrument into Spanish was carried out following cross-cultural translation procedures. First, the scale was translated from English into Spanish according to the parallel back-translation procedure, in which a bilingual person translates the scale from its original language to the language under study. Another bilingual individual, who is unfamiliar with the original scale, re-translates this version back to the original language. To ensure a correct translation and to avoid possible biases, the sequence just described was repeated twice, so four bilingual people carried out the parallel back-translation procedure, thus obtaining two pilot versions in Spanish for this study. Second, the items obtained were assessed by a committee formed from the individuals who participated in the translation process, and two psychology professors who selected the items that had maintained the original meaning and prepared the scale format and the instructions identically to the original version.

### 2.4. Body Composition Indicators

Anthropometric measurements (height, weight, and fat percentage) were carried out by trained evaluators in accordance with the protocol of the National Health and Nutrition Examination Survey (NHANES) [30]. The participants were weighed with a SECA 803^®^ scale, which has a precision of 100 g, in light clothing and barefoot. The participants were evaluated on the Frankfurt position using a SECA 213^®^ stadiometer (Seca, Hamburg, Germany) with a precision of 1 mm. The body mass index (BMI; kg/m^2^) was calculated using age- and sex-specific reference data from the World Health Organization [31]. Fat was estimated using the equation proposed by Slaughter [32]. For this, triceps and subscapular skinfolds were measured using a Lange^®^ adipometer (Bloomington, Minnesota, MN, USA) with a precision of 0.2 mm, and a constant pressure of 10 g/mm^2^.

### 2.5. Statistical Analysis

The descriptive statistics included means, standard deviations, and frequencies. The Kolmogorov–Smirnov test was applied to evaluate the data distribution. Differences between sexes were analyzed using a t-test for independent samples, and chi-square tests were used for categorical data.

Multilevel linear regression models were used to examine the associations between muscular strength and self-esteem. Our first model (model 1) was adjusted for age and sex. The second model (model 2) was additionally adjusted for age, sex, and BMI. This study also adjusted body fat to examine whether associations between muscular strength and self-esteem were independent of BMI. The third model (model 3) was controlled for age, sex, and body fat. This study also adjusted body fat to examine whether associations between muscular strength and self-esteem were independent from body fat. All analyses were performed with the SPSS V22 software (SPSS Inc., IBM Corp., Armonk, New York, NY, USA) [33].

## 3. Results

The sample included 1078 schoolchildren (598 boys and 480 girls). Table 1 presents the body composition indicators, muscular strength, and self-esteem data. Boys had a higher body weight and BMI than girls, and this difference was statistically significant. On the other hand, girls had higher body fat percentage values than boys; however, no differences were observed in age and height between boys and girls.

Boys had significantly higher muscular strength than girls, and the mean differences were as follows: 1.1 kg for upper limb strength, 12.3 cm for lower limb strength, and a 0.43 z-score for the general strength index. There were no significant differences between boys and girls for self-esteem (mean score; Table 1).

For boys and girls combined, Table 2 presents the results of the regression analysis describing the association between each value of muscular strength and self-esteem. Model 1 was adjusted for age and sex; model 2 for age, sex, and BMI; and model 3 for age, sex, and body fat. There were no significant associations between all muscular strength variables and self-esteem when adjusting for sex and BMI or body fat.

Table 3 and Table 4 present the results of the multilevel linear regression analysis describing the association between muscular strength variables and body composition indicators by sex. In boys (Table 3), there were positive associations (*p* < 0.05) between upper and lower limb strength and self-esteem when adjusting for age, BMI, and body fat. There were no significant associations between the general strength index and self-esteem when controlling for age and BMI or body fat.

In girls, muscular strength was not associated with self-esteem when the model was adjusted for age, BMI, or body fat. Upper and lower limb strength were positively associated with self-esteem, only when adjusting for age. The general strength index was not associated with self-esteem when adjusted for age, BMI, or body fat (Table 4).

## 4. Discussion

The aim of this study was to analyze the relationship of muscular strength from upper and lower limbs with self-esteem among Chilean schoolchildren, drawing independent associations with body composition indicators. For boys and girls combined, there were no significant associations between all muscular strength variables and self-esteem when adjusting for sex and BMI or body fat. In boys, there were positive associations between upper and lower limb strength with self-esteem when adjusting for age, body mass index, and body fat. On the other hand, muscular strength was not associated with self-esteem when controlling for age, body mass index, or body fat in girls.

Regarding the differences in muscular strength between boys and girls found in this study, these results are related to other studies that found higher values in children. In a study by Palacio-Agüero et al. [34], the 50th percentile for hand grip muscle strength ranged from 17.9 to 31.5 kg for boys and from 17.5 to 24.1 for girls. The differences were more marked after the age of 14. A study carried out with Chilean children showed the same differences in preadolescent children, with higher levels of strength [35]. For example, for children of an average age of 9 years old, the study found an average hand grip of 11.7 and 10.6 for boys and girls, respectively; while García-Hermoso et al. [35] (*n* = 2026) reported averages of 14.1 and 11.8, respectively, for the same age. These differences may be due to socioeconomic status, as our sample was drawn exclusively from public schools in the southern peripheral sector of Santiago, which is characterized by a low socio-economic level, while García-Hermoso’s sample was drawn from a central urban axis within the city, with a higher socioeconomic level. Previous evidence has shown a relationship between lower socioeconomic status and lower hand grip strength [36]. Among Spanish adolescents, similar values have been described, namely: 14.7 to 40.4 for boys and 13.6 to 24.6 for girls [37]. In a study carried out in North America, the mean grip strength (based on the combined maximum score of both hands) among young people aged 6 to 10, 11 to 14, and 15 to 19 years old was higher in boys than in girls for all age groups [38]. In relation to the strength of the lower limbs in long jump, in a study conducted by Yi Sun et al. [39], in Chinese boys and girls, the average values of the 50th percentile corresponded to 141.9 and 135.5, respectively, confirming the differences in levels of strength in this age group. Variability is attributed to body composition and maturation stage [40,41], whereas sex differences in strength may also reflect, in part, differences in the activity preferences of girls and boys [42].

Our results on muscle strength and self-esteem do not agree with those observed by Rodriguez-Ayllón et al. [43]. In their study, the authors reported a positive association between muscular strength and self-esteem in children, which was not modified by sex or weight status. In addition, Lubans et al. [44], in a study with adolescents, reported that muscular strength was associated positively with self-esteem (as a component of self-worth) in boys, but not in girls, similar to what was observed in our sample. Furthermore, in the Lubans et al. study, adiposity was inversely associated with self-esteem in girls, but not in boys. According to the authors, the explanation for such differences would be mediated by physical attractiveness, which is another component of self-worth. An important role of physical attractiveness in self-worth has been reported by other studies with young samples [45]. Thus, boys with more absolute strength, regardless of their BMI, showed more self-esteem; according to the authors, this because strength and muscularity are considered positive attributes in men of Western societies. In the case of girls, those with more absolute strength also had a higher BMI, which could be related to lower self-esteem. This is because girls can be influenced by the value attributed to the slim female image in Western societies.

A systematic review showed that most studies on self-esteem and its association with physical activity and fitness have been conducted in adolescents, with few studies done on children [39]. In any case, it could be considered that the reported associations between self-esteem and muscular strength may begin to manifest early among boys in our sample, while girls may have lower self-esteem due to a lower sense of physical attractiveness, which begins to manifest before adolescence. In addition, it must be considered that further studies are necessary in order to elucidate the direction of the effect [46].

### Strengths and Limitations

One of the strengths of the present study is the scope of its sample, which covered a representative group of the southern sector of Santiago, which allows for the generalization of the results. However, some methodological limitations must be considered. First, this study is cross-sectional, and therefore cannot assess the long-term trend of muscle strength development and self-esteem in Chilean boys and girls. Second, there are differences between children and adolescents in different areas of Chile in terms of their socioeconomic status, which can affect the variables of the study, and should lead to new studies aimed at determining the impact and relationship of socioeconomic variables with self-esteem and muscle strength. Third, self-esteem questionnaires were used as measures, which can lead to recall bias. Fourth, the general strength index as a variable did not achieve the expected impact and relationship within the study. By providing a unique Metropolitan Region dataset, the present study expanded the existing literature. In addition to identifying the relationship between muscle strength and self-esteem, which can be applied to public health policies and investments, our findings should be viewed as an opportunity to inform and motivate researchers to further examine these associations.

## 5. Conclusions

The present study demonstrates considerable associations between muscular strength (specifically in boys) and self-esteem among nine-year old Chilean schoolchildren, regardless of body composition indicators. The different associations found between the sexes have implications for future research, and may become interventions focused on improving muscular strength to promote mental health.

It is essential to consider self-esteem when exploring the associations between schoolchildren’s fitness and mental health. These aspects are necessary for health promotion programs aimed at improving physical fitness, which would have an effect beyond body weight, positively impacting physical and mental health in the young population and at later stages in life. In addition, the increase in self-esteem could predict the improvement of healthy behaviors and encourage the practice of physical activity, which is necessary for the improvement of muscular fitness, thus creating a virtuous circle that favors the health of children.

## Figures and Tables

**Table 1:** Descriptive analysis (mean (SD) or *n* (%)) of the sample.

Variables	Total (*n* = 1078)	Boys (*n* = 598)	Girls (*n* = 480)	*p*-Value
Age (years)	9.1 (1.1)	9.2 (1.2)	9.0 (1.1)	0.072 ^a^
Height (cm)	133.8 (9.1)	134.1 (9.0)	133.5 (9.3)	0.597 ^a^
Body weight (kg)	35.4 (10.5)	35.7 (11.3)	35.1 (9.4)	0.010 ^a^
Body mass index (kg/m^2^)	19.5 (3.9)	19.5 (4.2)	19.4 (3.6)	<0.001 ^a^
Body fat (%)	27.2 (9.3)	25.3 (9.9)	29.5 (8.1)	<0.001 ^a^
Upper limb strength (kg)	11.2 (4.0)	11.7 (4.0)	10.6 (3.9)	<0.001 ^a^
Lower limb strength (cm)	104.3 (20.5)	109.7 (21.1)	97.4 (17.5)	<0.001 ^a^
General strength index (z-score)	−0.04 (0.8)	0.19 (0.8)	−0.24 (0.7)	<0.001 ^a^
Self-esteem (mean score)	27.9 (3.9)	27.8 (3.8)	28.1 (4.0)	0.296 ^a^
Self-esteem (categorical)				0.009 ^b^
Low	275 (25.5)	148 (24.7)	127 (26.5)	
Middle	455 (42.2)	276 (46.2)	179 (37.3)	
High	348 (32.3)	174 (29.1)	174 (36.3)	

^a^ Value of significance of Student’s t-test for independent samples. ^b^ Value of significance of the Chi-square test.

**Table 2:** Adjusted analyses between the muscular strength of upper and lower limbs and self-esteem in 9-year old Chilean schoolchildren: independent associations with body composition indicators.

Muscular Strength	β Coefficient	95% CI	*p*-Value
Upper limb strength (kg) ^1^	−0.008	−0.066; 0.050	0.786
Upper limb strength (kg) ^2^	−0.007	−0.070; 0.054	0.798
Upper limb strength (kg) ^3^	0.005	−0.064; 0.055	0.879
Lower limb strength (cm) ^1^	0.012	−0.009; 0.014	0.687
Lower limb strength (cm) ^2^	0.024	−0.007, 0.016	0.453
Lower limb strength (cm) ^3^	0.020	−0.009; 0.016	0.565
General strength index (z-score) ^1^	−0.001	−0.281; 0.279	0.993
General strength index (z-score) ^2^	0.009	−0.249; 0.332	0.779
General strength index (z-score) ^3^	0.007	−0.257; 0.326	0.817

^1^ Basic adjustment: age and sex; ^2^ full adjustment: age, sex, and body mass index; ^3^ full adjustment: age, sex, and body fat; *p* < 0.05.

**Table 3:** Adjusted analyses between the muscular strength of upper and lower limbs and self-esteem in 9-year old Chilean boys: independent associations with body composition indicators.

Muscular Strength	β Coefficient	95% CI	*p*-Value
Upper limb strength (kg) ^1^	0.096	0.032; 0.159	0.002
Upper limb strength (kg) ^2^	0.067	0.011; 0.125	0.041
Upper limb strength (kg) ^3^	0.062	0.019; 0.116	0.047
Lower limb strength (cm) ^1^	0.095	0.022; 0.174	<0.001
Lower limb strength (cm) ^2^	0.091	0.025; 0.160	<0.001
Lower limb strength (cm) ^3^	0.090	0.039; 0.151	<0.001
General strength index (z-score) ^1^	0.019	−0.341; 0.380	0.078
General strength index (z-score) ^2^	0.022	−0.339; 0.382	0.089
General strength index (z-score) ^3^	0.010	−0.341; 0.384	0.075

^1^ Basic adjustment: age; ^2^ full adjustment: age and body mass index; ^3^ full adjustment: age and body fat; *p* < 0.05.

**Table 4:** Adjusted analyses between the muscular strength of upper and lower limbs and self-esteem in 9-year old Chilean girls: independent association with body composition indicators.

Muscular Strength	β Coefficient	95% CI	*p*-Value
Upper limb strength (kg) ^1^	0.067	0.029; 0.954	0.042
Upper limb strength (kg) ^2^	−0.025	−0.071; 0.122	0.601
Upper limb strength (kg) ^3^	−0.038	−0.054; 0.132	0.414
Lower limb strength (cm) ^1^	0.097	0.029; 0.184	0.031
Lower limb strength (cm) ^2^	−0.021	−0.070; 0.111	0.655
Lower limb strength (cm) ^3^	0.045	−0.055; 0.120	0.471
General strength index (z-score) ^1^	0.014	−0.409; 0.563	0.755
General strength index (z-score) ^2^	0.013	−0.418; 0.555	0.781
General strength index (z-score) ^3^	0.014	−0.410; 0.565	0.755

^1^ Basic adjustment: age; ^2^ full adjustment: age and body mass index; ^3^ full adjustment: age and body fat; *p* < 0.05.

## Data Availability

Not applicable.
